# Evaluation of the Antiproliferative Activity of the Leaves from *Arctium lappa* by a Bioassay-Guided Fractionation

**DOI:** 10.3390/molecules17021852

**Published:** 2012-02-14

**Authors:** Fabio Bahls Machado, Rafael Eidi Yamamoto, Karine Zanoli, Samara Requena Nocchi, Cláudio Roberto Novello, Ivânia Teresinha Albrecht Schuquel, Cássia Mônica Sakuragui, Heinrich Luftmann, Tânia Ueda-Nakamura, Celso Vataru Nakamura, João Carlos Palazzo de Mello

**Affiliations:** 1 Post-Graduate Program in Pharmaceutical Sciences, State University of Maringá, Av. Colombo, 5790, BR-87020-900, Maringá, PR, Brazil; Email: famafarm@yahoo.com.br (F.B.M.); kazanoli@hotmail.com (K.Z.); samaranocchi@hotmail.com (S.R.N.); tunakamura@uem.br (T.U.-N.); cvnakamura@uem.br (C.V.N.); 2 Department of Basic Sciences of Health, State University of Maringá, Av. Colombo, 5790, BR-87020-900, Maringá, PR, Brazil; Email: thexuxs@gmail.com; 3 Department of Pharmacy, State University of Maringá, Av. Colombo, 5790, BR-87020-900, Maringá, PR, Brazil; Email: crnovello@uem.br; 4 Department of Chemistry, State University of Maringá, Av. Colombo, 5790, BR-87020-900, Maringá, PR, Brazil; Email: itaschuquel@uem.br; 5 Federal University of Rio de Janeiro, Av. Pedro Calmon, 550, BR-21941-901, Rio de Janeiro, RJ, Brazil; Email: cmsakura12@gmail.com; 6 Organic Chemistry Institute, University of Münster, Münster D-48149, Germany; Email: luftman@uni-muenster.de

**Keywords:** Asteraceae, *Arctium lappa*, antiproliferative activity, sesquiterpene lactones

## Abstract

*Arctium lappa* L. (Asteraceae) is used in folk medicine around the World, and shows several kinds of biological activity, particularly *in vitro* antitumor activity in different cell lines. This study evaluated the antiproliferative activity of the crude extract, semipurified fractions, and isolated compounds from the leaves of *A. lappa*, through bioassay-guided testing in Caco-2 cells. The crude extract was obtained with a 50% hydroethanolic extract and then partitioned with hexane, ethyl acetate, and *n*-butanol. The ethyl-acetate fraction (EAF) showed antiproliferative activity. This fraction was subjected to sequential column chromatography over silica gel to afford onopordopicrin (**1**), mixtures of **1** with dehydromelitensin-8-(4'-hydroxymethacrylate) (**2**), a mixture of **2** with dehydromelitensin (**3**), mixture of **1** with melitensin (**4**), dehydrovomifoliol (**5**), and loliolide (**6**). The compounds were identified by spectroscopic methods (NMR, MS) and comparison with literature data. This is the first description of compounds **2–5** from this species. The compounds tested in Caco-2 cells showed the following CC_50_ (µg/mL) values: **1**: 19.7 ± 3.4, **1** with **2**: 24.6 ± 1.5, **2** with **3**: 27 ± 11.7, **1** with **4**: 42 ± 13.1, **6** 30 ± 6.2; compound **5** showed no activity.

## 1. Introduction

Greater burdock, *Arctium lappa* L. (Asteraceae), was introduced from Japan to Brazil, where it is widely used in folk medicine [1]. The first report on its antitumor activity was provided by Foldeak and Dombradi [2]. Testing in prostate-cancer cells revealed that the methanol extract of burdock seeds showed activity against the LNCaP cell line. Bioassay-guided cytotoxicity fractionation isolated the compounds lappaol A, C, and F, arctignan E, and arctiin. Lappaols A, C, and F showed activity, with IC_50_ values of 8, 16, and 40 μg/mL, respectively [3]. The roots contain inulin [4] and flavonoids [5]; and the fruits contain lignan [6]. Sesquiterpene lactones in Asteraceae characteristically show cytotoxic, antitumor, and other activities [7]. Onopordopicrin (a sesquiterpene lactone) isolated from the leaves of *A. lappa* [8] showed an IC_50_ of 15 μmol/L by MTT and PTP assays against a cell line of promyelocytic leukemia (HL60) [9]; in another experiment, onopordopicrin inhibited the tumor necrosis factor [10]. This study used a bio-guided assay to evaluate the antiproliferative activity of the crude extract, semipurified fraction, and isolated compounds from leaves of *A. lappa*, against Caco-2 cells.

## 2. Results and Discussion

### 2.1. Extraction Yield

The defatted leaves yielded 119.2 g (4.13%) of a lipophilic fraction, which showed no effect on Caco-2 cells. The extract was prepared with the defatted leaves (2,766 g) to yield 21.8% (604 g) to give the crude extract (CE). The CE (575 g) was partitioned with *n*-hexane (NHF), ethyl acetate (EAF), and *n*-butanol (NBF) to yield fractions of 5.3 g (0.53%), 25.46 g (4.43%), and 60.37 g (10.5%), respectively, and 461.84 g (80.32%) of the aqueous fraction (AQF).

### 2.2. Antiproliferative Activity

In the search for new anti-cancer drugs, numerous plant derivatives and phytochemicals have been evaluated for the prevention of this disease [11]. In the present study, active compounds were isolated by means of bioassay-guided methods in Caco-2 tumor cells. The CE showed CC_50_ (µg/mL) of 347.5, NHF > 500, NBF > 500, and EAF 7.24; of the 22 subfractions obtained from EAF#3#2, only three were active against Caco-2 cells (CC_50_ µg/mL): Subfractions 12 (23.8), 13 (25.6), and 14 (25.0) (Table 1).

**Table 1:** CC_50_ (µg/mL) of the subfractions obtained from fraction EAF#3#2 in Caco-2 cells.

Subfractions	CC_50_ (µg/mL) ( *x* ± *sd*)
1	>100
2	>100
3	52 ± 15.5
4	64.5 ± 19.09
5	47 ± 26.9
6	66 ± 7.07
7	>100
8	>100
9	>100
10	>100
11	78 ± 4.2
12	23.8 ± 2.9
13	25.1 ± 4.3
14	25 ± 0.0
15	29.3 ± 3.2
16	29.9 ± 5.3
17	59.1 ± 12.9
18	73.5 ± 2.1
19	74 ± 2.8
20	74 ± 0.0
21	>100
22	>100

(*x* ± *sd*) mean ± standard deviation.

The compound onopordopicrin (**1**) shows promise against some tumor-cell lines, including those tested in this experiment. This compound, obtained from subfractions 13 and 14, showed CC_50_ of 19.7 ± 3.4 µg/mL against Caco-2 cells. Onopordopicrin was previously reported as cytotoxic *in vitro* against KB cell lines (human nasopharyngeal squamous cell carcinoma) [12]. This sesquiterpene lactone was the major component of the fractions, and was isolated in all the most active fractions and in considerable quantity, especially in fraction 13 (95.6 mg). This suggests that the compound responsible for the antiproliferative activity of the extract on the Caco-2 cell line is onopordopicrin (**1**).

Savina *et al*. [13] determined that the onopordopicrin content in the ethyl-acetate fraction from the juice of leaves of *A. lappa* is in the range of 0.035–0.005%, with a total sesquiterpene content of approximately 0.01%. In this study, the isolated compound comprised 0.02% (118.4 mg) of CE (575 g). Therefore, compound **1** is appropriate for use in studies to validate the analytical methodology, to determine its mechanism of action, and to evaluate its toxicity, and can also be used as a biological marker. Further, detailed evaluation is needed; Rocha [14] noted reservations regarding the oral administration of sesquiterpene lactones for therapeutic purposes, because of their toxicity.

Subfraction 12, with a mixture of onopordopicrin (**1**) and dehydromelitensin-8-(4'-hydroxy-methacrylate) (**2**), showed a CC_50_ of 24.6 ± 1.5 µg/mL. This activity is probably related to the presence of onopordopicrin (**1**), as the samples containing onopordopicrin showed similar results to the EAF.

In subfraction 13, the mixture containing dehydromelitensin-8-(4'-hydroxymethacrylate) (**2**) and dehydromelitensin (**3**) showed a CC_50_ value of 27 ± 11.7 µg/mL; onopordopicrin (**1**) in greater quantity with melitensin (**4**, 3.4 mg) showed a value of 42 ± 13.1 µg/mL. The compound dehydrovomifoliol (**5**) showed no antiproliferative activity at concentrations below 100 µg/mL. Dehydrovomifoliol was previously evaluated for its cytotoxic activity against the cancer cell lines Hone-1 (human nasopharyngeal carcinoma), KB (oral squamous cell carcinoma), and HT29 (colorectal carcinoma) using methylene blue and the anticancer drugs etoposide and cisplatin, both as a positive control, and showed a cytotoxic activity of 4.8 with CC_50_ of 4.0 and 5.7 µmol/L, respectively [15]. Loliolide (**6**) showed a CC_50_ of 30 ± 6.2 µg/mL. In this study, the compounds dehydromelitensin-8-(4'-hydroxy-methacrylate) (**2**), dehydromelitensin (**3**), melitensin (**4**), and dehydrovomifoliol (**5**) were isolated from the leaves of *A. lappa* for the first time.

## 3. Experimental

### 3.1. General

NMR spectra were recorded in CDCl_3_ and/or CD_3_OD/CDCl_3_ at ambient temperature, with TMS as the internal standard, in a Varian Mercury Plus 300 BB, 300 MHz. The MS spectra were obtained by HR-ESI-TOF of MicroTof (Bruker-Daltonics, Bremen, Germany) at the Organic Chemistry Institute of Münster University, Germany.

### 3.2. Plant Material

Leaves of *Arctium lappa* L. were collected in February 2008 (17.3 kg) at the Dois Irmãos Farm in Marialva, Paraná, Brazil (S23°27'47.0"; W051°45'52.0"; altitude 523 m a.s.l.). A voucher specimen is deposited in the Herbarium of the Instituto de Biologia of the Universidade Federal do Rio de Janeiro, under number RFA 35777. The species was identified by Prof. Dr. Cássia Mônica Sakuragui, by comparison with the nomenclatural type. After drying, the leaves were ground in a hammer mill (Tiger ASN-5).

### 3.3. Extraction, Isolation, and Identification of Compounds

Air-dried leaves (VD; 2,886 g) were defatted by dynamic maceration with *n-*hexane 1:5 (w/v; 8 h). The VD (2,766 g) was extracted by turbo-extraction (Ultra-Turrax® UTC115KT) for 15 min at ≤40 °C in EtOH/H_2_O (1:1) at 10% (w/v). Next, the crude extract (CE) was filtered and concentrated in a rotavapor under reduced pressure and then lyophilized, for a yield of 604 g. The CE (575 g) was dissolved in water (5,750 mL) and partitioned with *n*-hexane (NHF), ethyl acetate (EAF), and *n*-butanol (NBF), 12 times in each solvent, until no coloration was observed in the organic phase, affording the aqueous fraction (AQF). The EAF (22 g) was subjected to vacuum liquid chromatography (VLC; column: h: 200 mm Ø 65 mm), employing silica gel 60 (0.063–0.200 mm; Merck, Darmstadt, Germany), and as eluents: Hex (880 mL; 0.05 g), CH_2_Cl_2_ (1,100 mL; 0.53 g), EtOAc (1,780 mL; 12.7 g), MeOH (1,870 mL; 7.7 g), and MeOH:H_2_O (1:1; 1,760 mL; 0.59 g). The ethyl-acetate subfraction (12.7 g) was subjected to a new VLC using the previous conditions with the eluent system: CHCl_3_ (1,350 mL), CHCl_3_:MeOH (8:2; 600 mL), CHCl_3_:MeOH (6:4; 450 mL), MeOH (525 mL), and *n*-BuOH (1760 mL). The CHCl_3_:MeOH (8:2) subfraction (EAF#3#2; 4.7 g) was subjected to a new VLC using: Hex:EtOAc (6:4; 1,000 mL), Hex:EtOAc (55:45; 500 mL), Hex:EtOAc (1:1; 900 mL), Hex:EtOAc:MeOH (50:49:1; 400 mL), Hex:EtOAc:MeOH (50:48:2; 200 mL), Hex:EtOAc:MeOH (50:45:5; 2,200 mL), Hex:EtOAc:MeOH (5:4:1; 200 mL), Hex:EtOAc:MeOH (5:3:2; 200 mL), EtOAc:MeOH (1:1; 300 mL), and MeOH (400 mL). These fractions were assembled by chromatographic similarity to yield 22 subfractions, which were concentrated under reduced pressure in a rotavapor and then lyophilized. Analytical TLC was carried out on precoated aluminum sheets (Kieselgel 60 F_254_; Merck) with Hex:EtOAc:MeOH (5:4:1).

**Figure 1:**
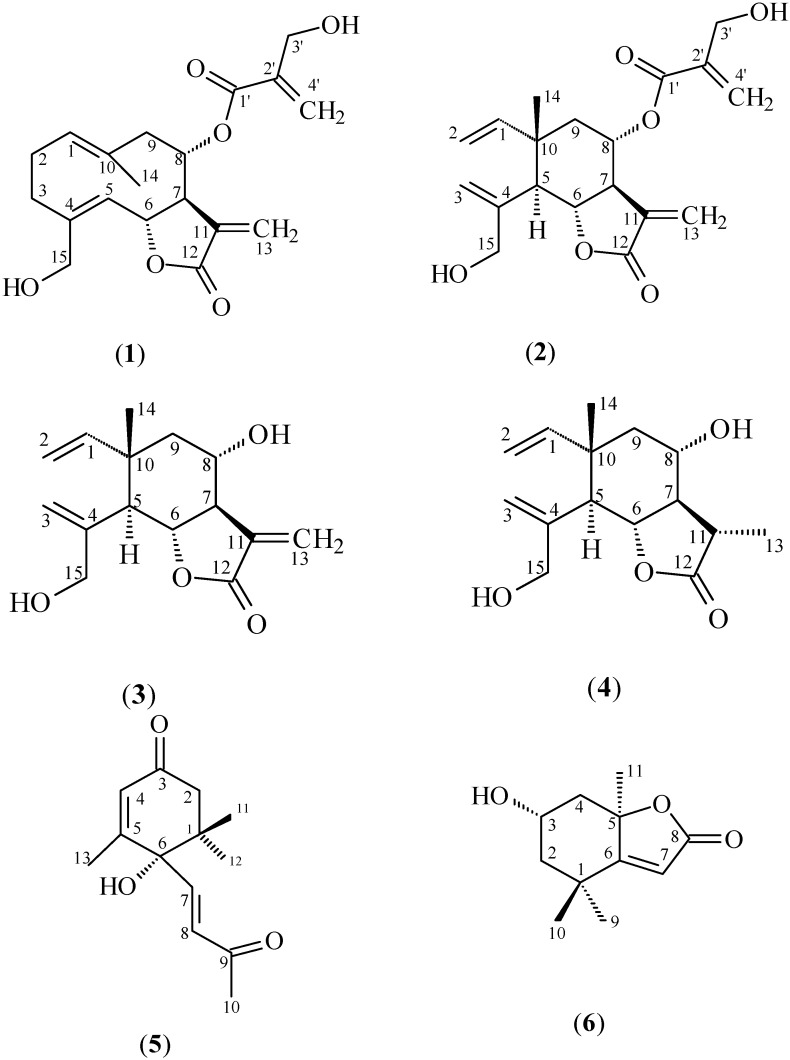
Compounds isolated from the leaves of *Arctium lappa*: onopordopicrin (**1**), dehydromelitensin-8-(4'-hydroxymethacrylate) (**2**), dehydromelitensin (**3**), melitensin (**4**), dehydrovomifoliol (**5**), and loliolide (**6**).

The 22 subfractions were evaluated by bio-guided assay in Caco-2 tumor cells. The subfractions 12 (898 mg), 13 (636 mg), and 14 (269 mg) were submitted to flash chromatography with silica gel (0.040–0.063 mm; Merck, Darmstadt, Germany; column: h: 350 mm Ø 15 mm), and as eluents: Hex:EtOAc (6:4), Hex:EtOAc (58:42), Hex:EtOAc (55:45), Hex:EtOAc (1:1), Hex:EtOAc:MeOH (5:4:1), and MeOH. Subfraction 12 yielded onopordopicrin (**1**) and dehydromelitensin-8-(4'-hydroxymethacrylate) (**2**) (116.7 mg); subfraction 13: **1** (95.6 mg), and a mixture of **2** with dehydromelitensin (3, 18.8 mg), mixture of **1** with melitensin (4, 3.4 mg), dehydrovomifoliol (5, 3.5 mg), and loliolide (6, 4.0 mg); subfraction 14: **1** (22.8 mg) (Figure 1). The compounds were identified by NMR 1D and 2D, HR-ESI-TOF-MS, and by comparison with the literature data [16,17,18,19,20].

### 3.4. Cell Culture

For the experiments we used Caco-2 cells (human intestinal epithelial cells, derived from colorectal carcinoma). They were grown in DMEM (Dulbecco's Modified Eagle Medium, Gibco Invitrogen Corporation, Grand Island, NY, USA) supplemented with 10% fetal bovine serum (FBS, Gibco) and 50 μg/mL gentamicin at 37 °C with 5% CO_2_ (Fisher Scientific, Isotemp, Asheville, NC, USA).

### 3.5. Antiproliferative Activity Assay

The antiproliferative activity was assessed by sulforhodamine colorimetric assay according to Skehan *et al*. [21]. A suspension of 5 × 10^5^ cells/mL of Caco-2 cells in DMEM medium supplemented with 10% FBS and 50 µg/mL of gentamicin was added to each well in a 96-well microplate (TPP^®^). The plates were incubated in a 5% CO_2_-air mixture at 37 °C to obtain confluent growth of the cells. After 24 h, the medium was removed, and one of several concentrations of the crude extracts, fractions, or isolated compounds (1, 5, 10, 25, 50, 100, 200, and 500 µg/mL) was added to each well containing the cells, and the plates were incubated for 48 h. The nonadherent cells were removed by washing with DMEM medium, and the adhered cells were fixed with 50 µL/well of 10% trichloroacetic acid (Synth^®^) at 4 °C for 1 h; after that, they were washed with water, and 50 µL/well of sulforhodamine B (0.4% w/v) was added; the microplate was then maintained at 4 °C for 30 min. Next, the sulforhodamine B was removed, and the microplate was washed four times with 1% acetic acid; then, 150 µL/well of 10 mmol/L unbuffered Tris-base solution (Sigma Chemical Co., St Louis, MO, USA) was added. Next, the absorbance of each individual well was read in a 96-well plate reader (BIOTEK Power Wave XS) at 530 nm. Each experiment was performed in triplicate on three different occasions. The antiproliferative activity was determined according to the following formula: % cell destruction = 1 − (ODtc/ODcc), where: ODtc = optical density of the treated cells and ODcc = optical density of the control cells.

### 3.6. Statistical Analysis

The statistical analysis was performed using GraphPad Prism (GraphPad Software Inc., La Jolla, CA, USA). The results were expressed as mean ± standard deviation and were analyzed using one-way ANOVA. Significant differences were determined by Tukey test, with *p* < 0.05 as the criterion for significance.

## 4. Conclusions

The compound onipordopicrin (**1**) is the main compound in leaves of *Arctium lappa* L., and demonstrated antiproliferative activity against Caco-2 cells. In addition, this compound can be used as a biological and chemical marker for this species.

## Supplementary Materials

Supplementary materials can be accessed at: http://www.mdpi.com/1420-3049/17/2/1852/s1.
