# Supplementary material for: Evaluation of the Antiproliferative Activity of the Leaves from Arctium lappa by a Bioassay-Guided Fractionation
Source: Molecules. 2012 Feb 14;17(2):1852–9. doi: 10.3390/molecules17021852 (PMC6268082; doi:10.3390/molecules17021852)
Supplement: Supplementary file 1 [file molecules-17-01852-s001.doc]

**Supplementary Information**

**Evaluation of the Antiproliferative Activity of the Leaves from *Arctium* *lappa* by a Bioassay-Guided Fractionation**

Fábio Bahls Machado ^1^, Rafael Eidi Yamamoto ^2^, Karine Zanoli ^1^, Samara Requena Nocchi ^1^, Cláudio Roberto Novello ^3^, Ivânia Teresinha Albrecht Schuquel ^4^, Cássia Mônica Sakuragui ^5^, Heinrich Luftmann ^6^, Tânia Ueda-Nakamura ^1^, Celso Vataru Nakamura ^1^ and
João Carlos Palazzo de Mello ^1^,*

^1^ Programa de Pós-Graduação em Ciências Farmacêuticas, Universidade Estadual de Maringá,
Av. Colombo, 5790, BR-87020-900, Maringá, PR, Brazil; E-Mails: famafarm@yahoo.com.br (F.B.M.); kazanoli@hotmail.com (K.Z.); samaranocchi@hotmail.com (S.R.N.);
tunakamura@uem.br (T.U.-N.); cvnakamura@uem.br (C.V.N.)

^2^ Departamento de Ciências Básicas da Saúde, Universidade Estadual de Maringá, Av. Colombo, 5790, BR-87020-900, Maringá, PR, Brazil; E-Mail: thexuxs@gmail.com

^3^ Departamento de Farmácia, Universidade Estadual de Maringá, Av. Colombo, 5790,
BR-87020-900, Maringá, PR, Brazil; E-Mail: crnovello@uem.br

^4^ Departamento de Química, Universidade Estadual de Maringá, Av. Colombo, 5790, BR-87020-900, Maringá, PR, Brazil; E-mail: itaschuquel@uem.br

^5^ Universidade Federal do Rio de Janeiro, Av. Pedro Calmon, 550, BR-21941-901, Rio de Janeiro, RJ, Brazil; E-Mail: cmsakura12@gmail.com

^6^ Organisch-Chemisches Institut, Universität Münster, D-48149, Münster, Germany;

E-Mail: luftman@uni-muenster.de

***** Author to whom correspondence should be addressed; E-Mail: mello@uem.br;
Tel.: +55-44-3011-4816; Fax: +55-44-3011-5050.

**Figure 1S.** ^1^H-NMR spectrum of compound **1** (CDCl_3_/CD_3_OD, 300 MHz).


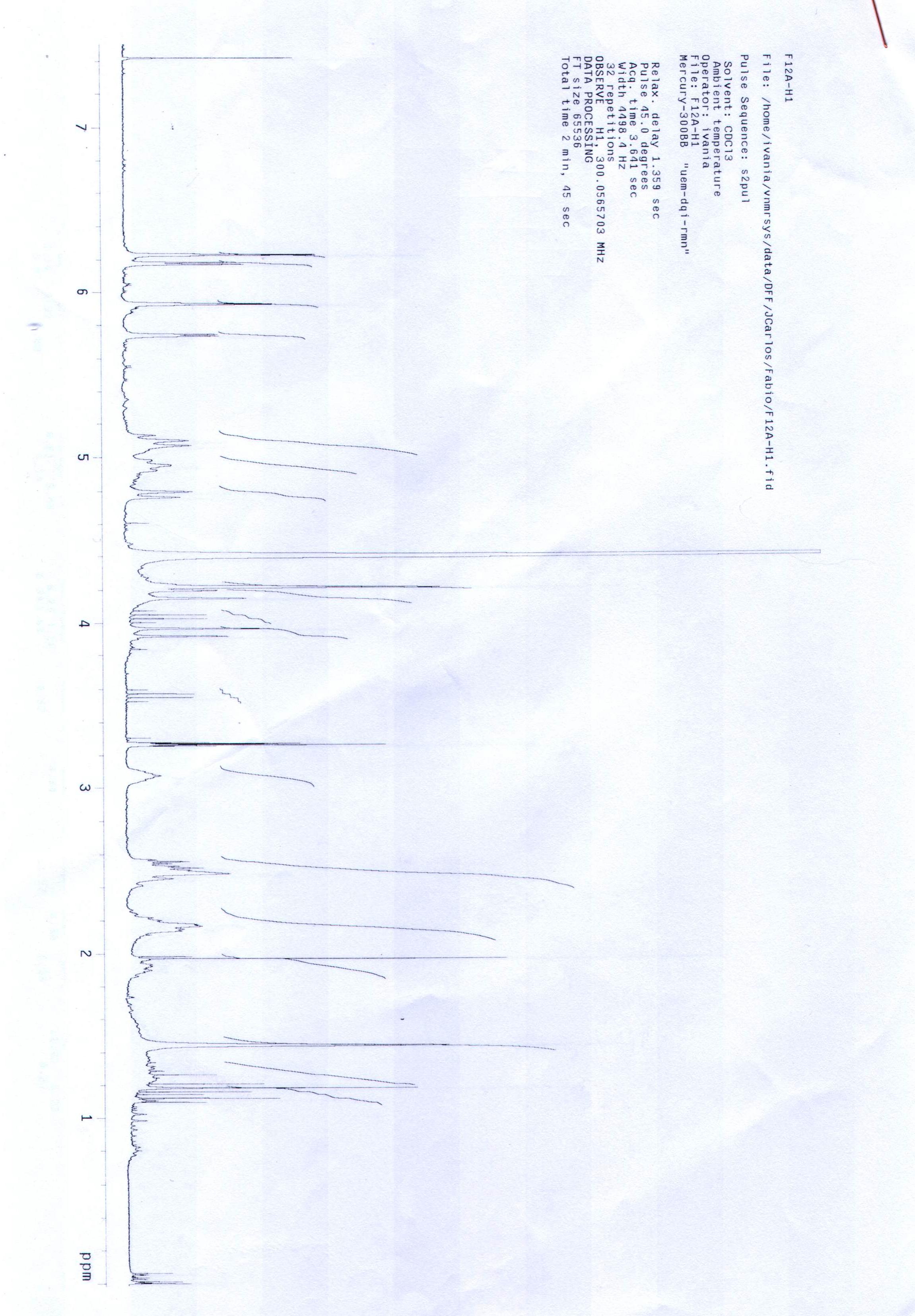


**Onopordopicrin (1)**

**Table 1S.** Data of NMR (CDCl_3_/CD_3_OD, 300 MHz for ^1^H and 75 MHz for ^13^C) of onopordopicrin (**1**) [17].

| **C** | **δ^13^C/DEPT** | **gHSQC**  **δ^1^H (nH; m, *J* = Hz)** | **gCOSY**  **^1^H×^1^H** | **gHMBC**  **^1^H→^13^C** |
| --- | --- | --- | --- | --- |
| 1 | 129.6/CH | 4.96 (1H; t; 9.0) | H_14_; H_2_ | C_3_; C_9_; C_14_ |
| 2 | 25.8/CH_2_ | 2.24-2.10 (2H; m) | H_3a,b_; H_1_ | C_3_; C_4_; C_10_ |
| 3 | 34.1/CH_2_ | 2.54 (1H; dt; 11.7; 3.6)  1.91 (1H; m) | H_3b_; H_2ab_  H_3a_; H_2a,b_ | C_2_; C_4_  C_2_; C_4_ |
| 4 | 144.4/C | - | - | - |
| 5 | 127.9/CH | 4.78 (1H; d; 9.6) | H_6_ | C_7_; C_15_ |
| 6 | 77.1/CH | 5.11 (1H; d;, 9.6) | H_5_ |  |
| 7 | 52.8/CH | 3.07 (1H; m) | H_8_; H_13a,b_ |  |
| 8 | 72.8/CH | 5.08 (1H; m) | H_7_; H_8a,b_ |  |
| 9 | 48.7/CH_2_ | 2.48-2.41 (2H; m) | H_8_ | C_1_; C_7_; C_8_; C_10_; C_14_ |
| 10 | 131.8/C | - | - | - |
| 11 | 135.3/C | - | - | - |
| 12 | 170.6/C | - | - | - |
| 13 | 125.2/CH_2_ | 5.74 (1H; d; 2.7)  6.18 (1H; d; 3.3) | H_7_  H_7_ | C_7_; C_12_  C_7_; C_11_; C_12_ |
| 14 | 16.3/CH_3_ | 1.44 (3H; s) | - | C_1_; C_9_; C_10_ |
| 15 | 60.0/CH_2_ | 3.95 (1H; dd; 14.1; 0.7)  4.18 (1H; dd; 14.1; 1.0) | H_15b_  H_15a_ | C_3_; C_4_; C_5_  C_3_; C_4_; C_5_ |
| 1' | 165.0/C | - | - | - |
| 2' | 139.8/C | - | - | - |
| 3' | 60.4/CH_2_ | 4.22 (2H; q; 1.5) | H_4’a,b_ | C_1’_; C_4’_ |
| 4' | 125.2/CH_2_ | 5.93 (1H; q; 1.5)  6.23 (1H; q; 1.5) | H_3’_; H_4’b_  H_3’_; H_4’a_ | C_1’_; C_2’_; C_3’_  C_1’_; C_2’_; C_3’_ |

**Figure 2S.** ^1^H-NMR spectrum of compound **2** (CDCl_3_/CD_3_OD, 300 MHz).

**
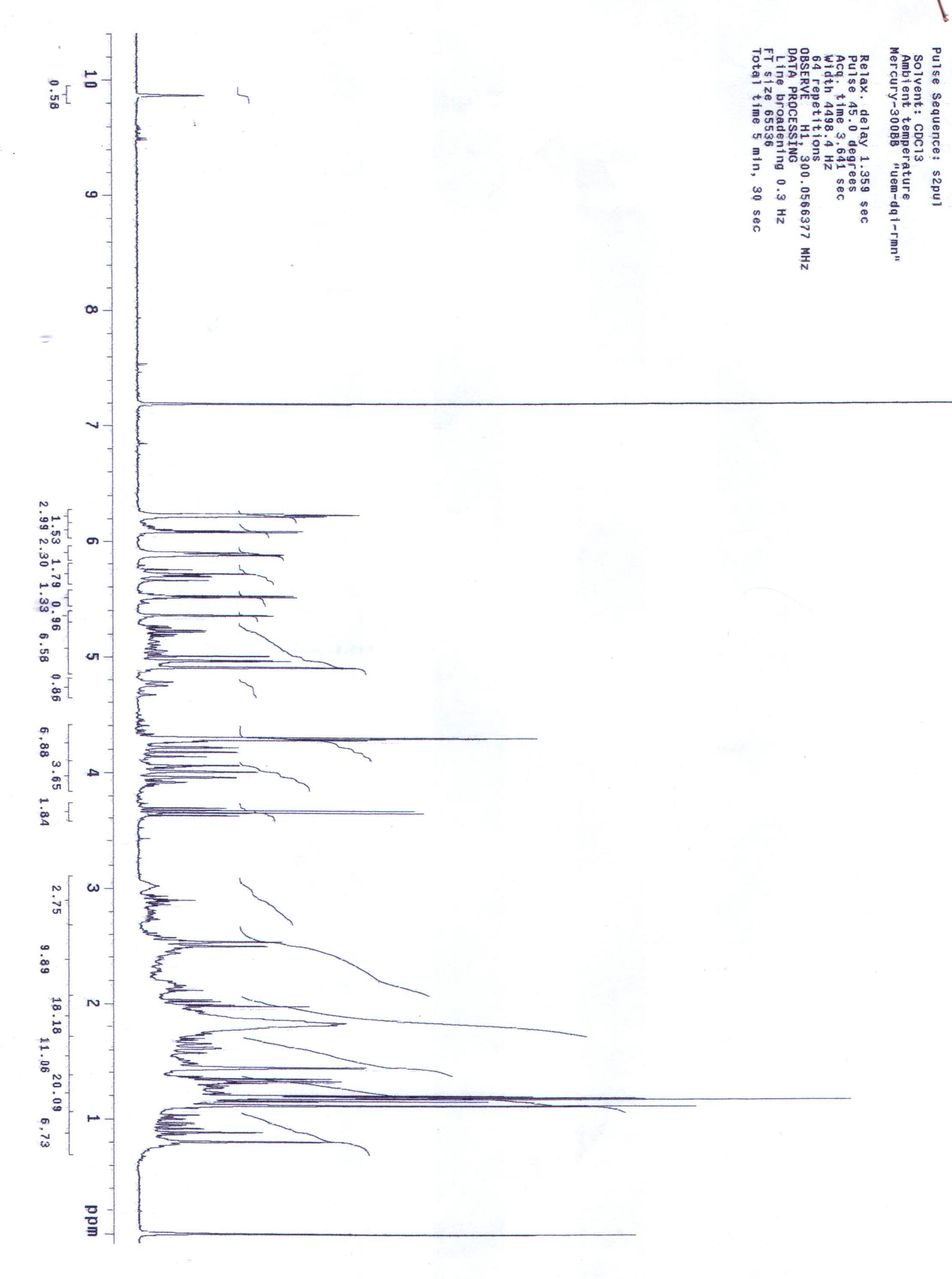
**

**Dehydromelitensin-8-(4'-hydroxy-methacrylate) (2)**

**Table 2S.** Data of NMR (CDCl_3_/CD_3_OD, 300 MHz for ^1^H and 75 MHz for ^13^C) of dehydromelitensin-8-(4'-hydroxy-methacrylate) (**2**) [18].

| **C** | **δ^13^C/DEPT** | **gHSQC**  **δ^1^H (nH; m, *J* = Hz)** | **gCOSY**  **^1^H×^1^H** | **gHMBC**  **^1^H→^13^C** |
| --- | --- | --- | --- | --- |
| 1 | 145.8/CH | 5.70 (1H; dd; 17.4; 10.8) | H2_a,b_ | C_4_ |
| 2 | 113.4/CH_2_ | α 4.99 (1H; d; 10.8)  β 4.94 (1H; d; 17.4) | H_1_  H_1_ | C_1_; C_9_  C_1_; C_9_ |
| 3 | 115.4/CH_2_ | α 5.36 (1H; t; 1.5)  β 4.91 (1H; sl) |  | C_5_  C_5_ |
| 4 | 143.9/C | - | - |  |
| 5 | 50.8/CH | 2.51 (1H; d; 11.7) | H_6_ | C_3_; C_4_; C_6_; C_7_; C_10_; C_14_ |
| 6 | 78.9/CH | 4.18 (1H; t; 11.7) | H_5_; H_7_ |  |
| 7 | 52.6/CH | 2.90 (1H; tt; 10.8; 3.0) | H_13a.b_; H_6_; H_8_ |  |
| 8 | 69.9/CH | 5.23 (1H; td; 10.8; 4.2) | H_9β_; H_7_ |  |
| 9 | 45.3/CH_2_ | α 1.61 (1H; dd; 12.7; 10.8)  β 2.00 (1H; dd; 12.7; 4.2) | H_9β_; H_8_  H_9α_; H_8_ | C_1_; C_8_  C_5_; C_8_ |
| 10 | 41.9/C | - | - | - |
| 11 | 136.8/C | - | - | - |
| 12 | 169.4/C | - | - | - |
| 13 | 120.4/CH_2_ | 6.09 (1H; d; 3.0)  5.52 (1H; d; 2.7) | H_13b_; H_7_  H_13a_; H_7_ | C_7_; C_12_ |
| 14 | 18.9/CH_3_ | 1.12 (3H; s) | - | C_1_; C_5_; C_9_; C_10_ |
| 15 | 67.6/CH_2_ | 4.04 (1H; d; 14.4)  3.99 (1H; d; 14.4) | H_15b_; H_3α_  H_15a_ | C_3_; C_4_  C_3_; C_4_; C_5_ |
| 1' | 165.5/C | - | - | - |
| 2' | 139.4/C | - | - | - |
| 3' | 62.5/CH_2_ | 4.30 (2H; t; 1.2) | H_4’a,b_ | C_1’_; C_2’_; C_4’_ |
| 4' | 126.9/CH_2_ | 6.23 (1H; m)  5.88 (1H; m) | H_3’_; H_4’b_  H_3’_; H_4’a_ | C_1’_; C_2’_; C_4’_  C_1’_; C_2’_; C_4’_ |

**Figure 3S.** ^1^H-NMR spectrum of compound **3** (CDCl_3_/CD_3_OD, 300 MHz).

**
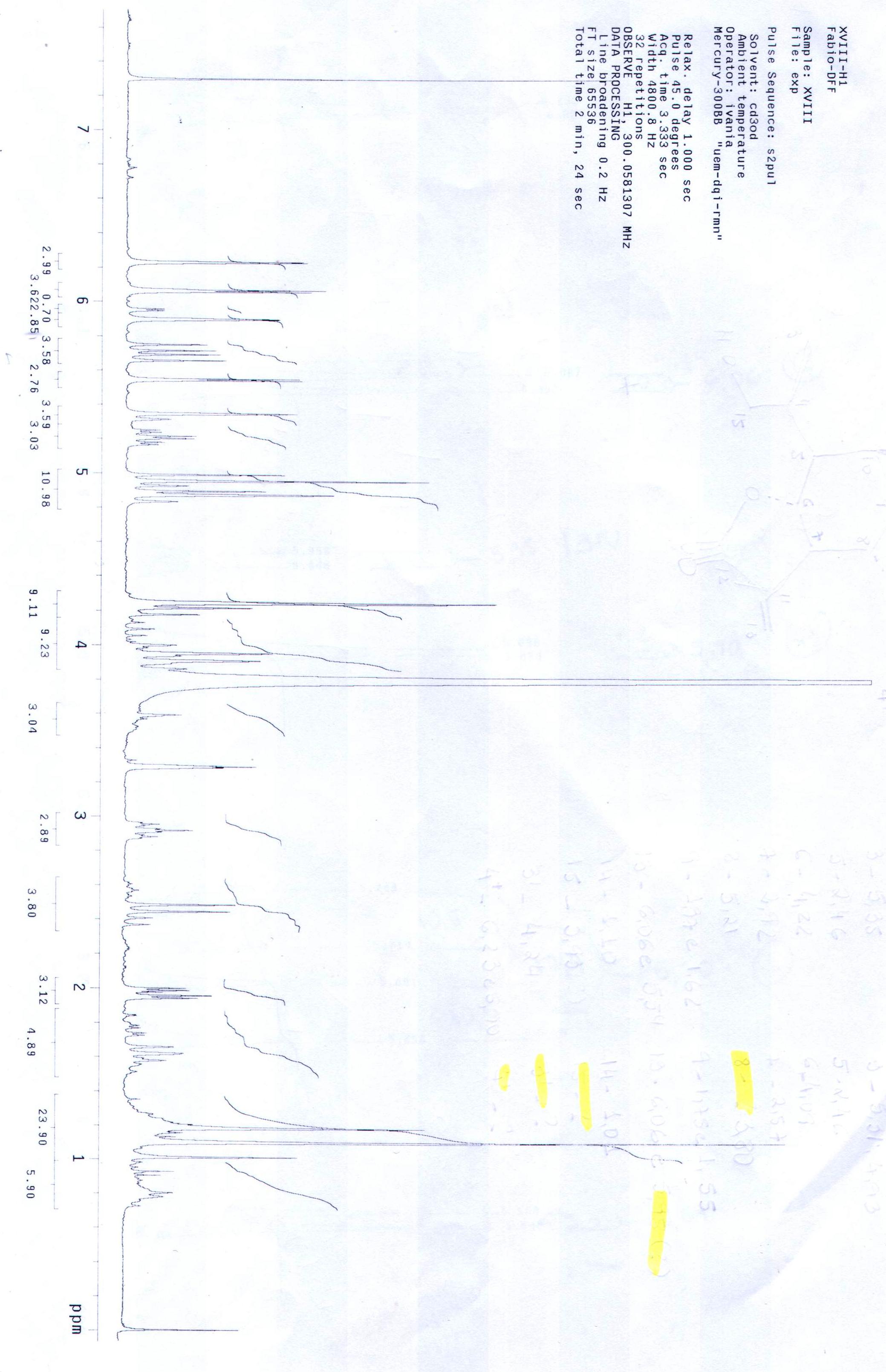
**

**Dehydromelitensin (3)**

**Table 3S.** Data of NMR (CDCl_3_/CD_3_OD, 300 MHz for ^1^H and 75 MHz for ^13^C) of dehydromelitensin (**3**) [12].

| **C** | **δ^13^C/DEPT** | **gHSQC**  **δ^1^H (nH; m, *J* = Hz)** | **gCOSY**  **^1^H×^1^H** | **gHMBC**  **^1^H→^13^C** |
| --- | --- | --- | --- | --- |
| 1 | 146.6/CH | 5.70 (1H; dd; 17.4; 10.5) |  | C_10_ |
| 2 | 111.8/CH_2_ | α 4.97 (1H; m)  β 4.83 (1H; m) |  | C_1_ |
| 3 | 114.4/CH_2_ | α 5.31 (1H; sl)  β 4.93 (1H; sl) | H_3β_  H_3α_ | C_15_  C_15_ |
| 4 | 144.2/C | - | - |  |
| 5 | 50.5/CH | 2.39 (1H; d; 11.7) | H_6_ | C_1_; C_4_; C_6_; C_15_ |
| 6 | 79.6 /CH | 4.09 (1H; t; 11.7) | H_5_; H_7_ | C_10_; C_11_ |
| 7 | 55.1/CH | 2.75 (1H; tt; 10.8; 3.0) | H_13a.b_; H_6_; H_8_ |  |
| 8 | 66.7/CH | 3.80 (1H; m) | H_9β_; H_7_; H_9α_ |  |
| 9 | 49.3/CH_2_ | α 1.55 (1H; dd; 13.5; 10.5)  β 1.75 (1H; dd; 13.5; 4.2) | H_9β_; H_8_  H_9α_; H_8_ |  |
| 10 | 41.8/C | - | - | - |
| 11 | 137.9/C | - | - | - |
| 12 | 170.1/C | - | - | - |
| 13 | 120.5/CH_2_ | 5.95 (1H; d; 2.4  6.07 (1H; d; 3.0) | H_13b_; H_7_  H_13a_; H_7_ |  |
| 14 | 18.7/CH_3_ | 1.01 (3H; s) | - | C_1_; C_9_; C_10_ |
| 15 | 66.5/CH_2_ | 3.93 (2H, m) |  |  |

**Figure 4S.** ^1^H-NMR spectrum of compound **4** (CDCl_3_/CD_3_OD, 300 MHz).

**
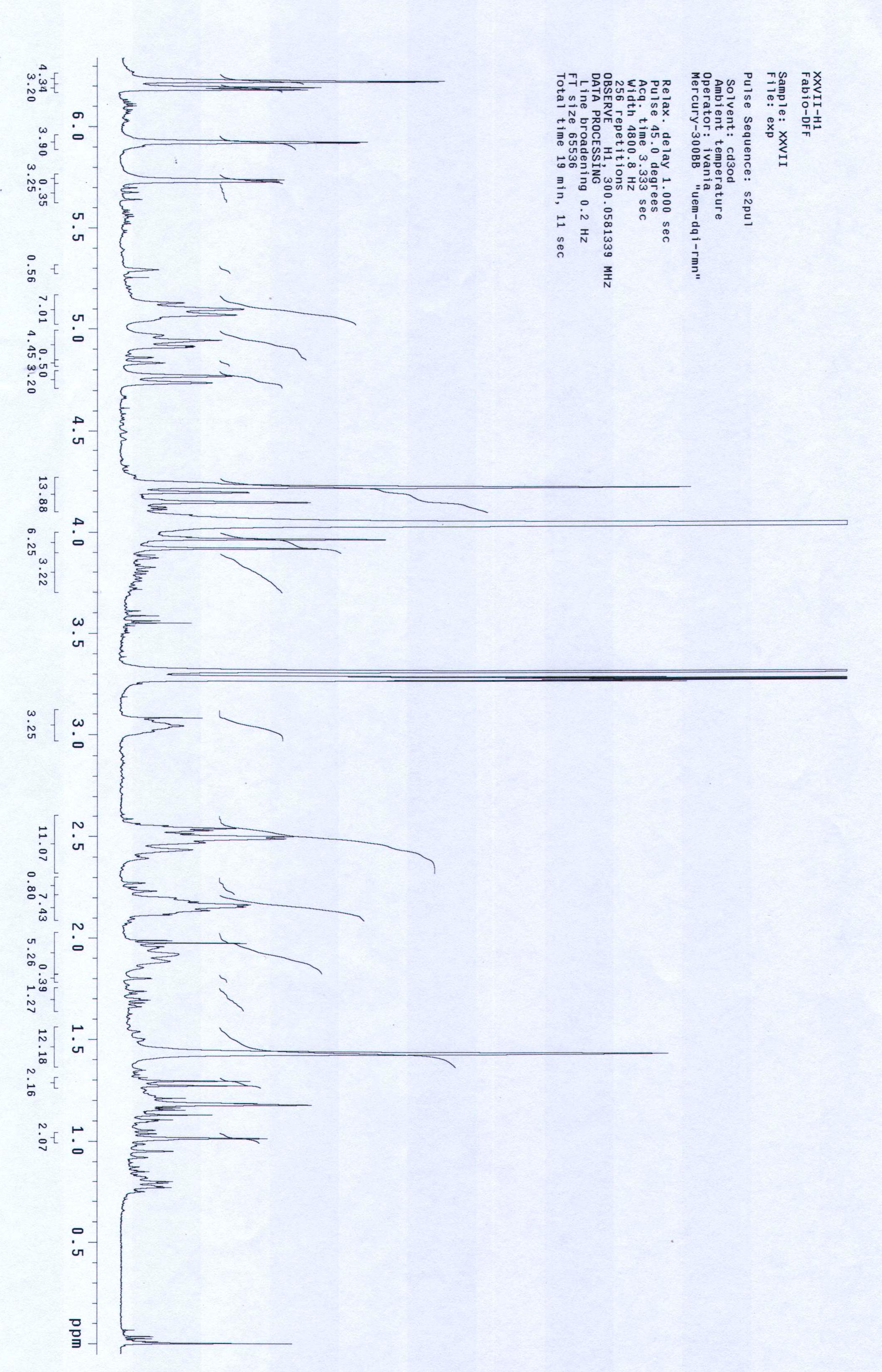
**

**Melitensin (4)**

**Table 4S.** Data of NMR (CDCl_3_/CD_3_OD, 300 MHz for ^1^H and 75 MHz for ^13^C) of melitensin (**4**) [13].

| **C** | **δ^1^H (nH; m, *J* = Hz)** | **gCOSY**  **^1^H×^1^H** | **Melitensin [13]*** |
| --- | --- | --- | --- |
| 1 | 5.69 (1H; dd; 17.4; 10.8) | H_2α_; H_2β_ | 5.75 (dd; 17; 11) |
| 2 | α 4.93(1H; m)  β 4.89 (1H; d; 17.4) | H_1_  H_1_ | 5.05 (d; 11)  5.01 (d; 17) |
| 3 | α 5.29 (1H; sl)  β 4.83 (1H; sl) | H_3β_  H_3α_ | 5.38 (s)  4.95 (s) |
| 5 | 2.25 (1H; d; 11.7) | H_6_ | 2.40 (d; 11) |
| 6 | 4.15 (1H; m) | H_5_; H_7_ | 4.15 (t; 11) |
| 7 | 1.70 (1H; m)) | H_11_; H_6_; H_8_ | 1.85 (m) |
| 8 | 3.78 (1H; ddd; 10.5; 10.2; 4.2) | H_9β_; H_7_; H_9α_ | 3.95 (brddd; 10.5; 5.3) |
| 9 | α 1.50 (1H; dd; 13.2; 10.8)  β 1.71 (1H; dd; 13.2; 4.2) | H_9β_; H_8_  H_9α_; H_8_ | 1.62 (d; 12)  1.80 (m) |
| 11 | 2.59-2.50 (1H; m) | H_7_; H_13_ | 2.62 (dq; 12; 7) |
| 13 | 1.29 (3H; d; 6.9) | H_11_ | 1.41 (d; 7) |
| 14 | 1.02 (3H; s) | - | 1.11 (s) |
| 15 | 4.03 (1H, m)  3.85 (1H; d; 13.8) |  | 4.08 (d; 14  3.98 (d; 14) |

**Figure 5S.** ^1^H-NMR spectrum of compound **5** (CDCl_3_/CD_3_OD, 300 MHz).

**
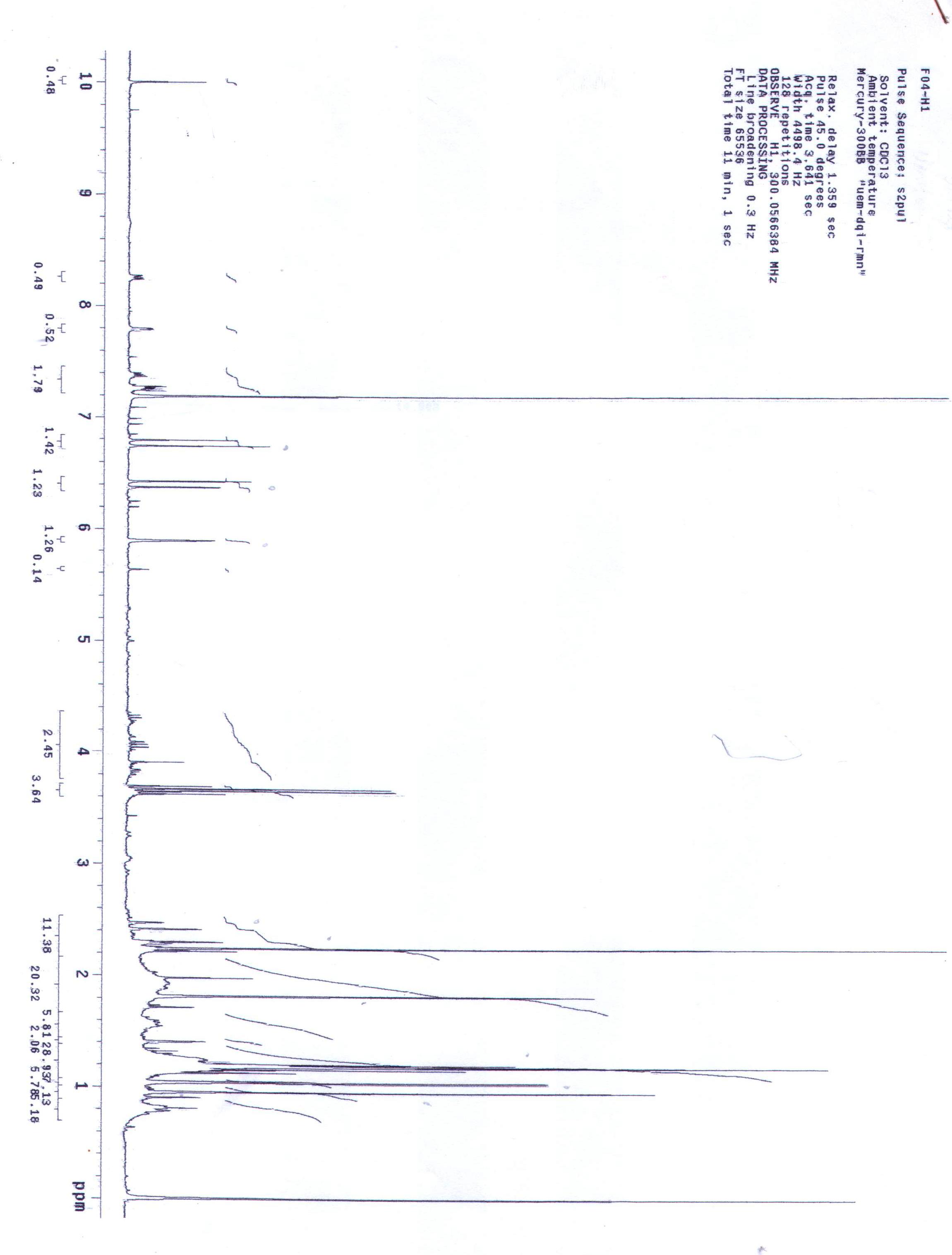
**

**Dehydrovomifoliol (5)**

**Table 5S.** Data of NMR (CDCl_3_/CD_3_OD, 300 MHz for ^1^H and 75 MHz for ^13^C) of dehydrovomifoliol (**5**) [14].

| **C** | **δ^13^C/DEPT** | **gHSQC**  **δ^1^H (nH; m, *J* = Hz)** | **gCOSY**  **^1^H×^1^H** | **gHMBC**  **^1^H→^13^C** |
| --- | --- | --- | --- | --- |
| 1 | 41.6/C | - | - | - |
| 2 | 49.8/CH_2_ | 2.44 (1H; d; 17.1)  2.27 (1H; dd; 17.1; 1.2) | H_2b_  H_2a_ | C_3_; C_6_; C_11_  C_3_ |
| 3 | 197.1/C | - | - | - |
| 4 | 128.1/CH | 5.89 (1H; quint; 1.2) | H_13_ |  |
| 5 | 160.4/C | - | - | - |
| 6 | *79.6/C | - | - | - |
| 7 | 145.1/CH | 6.77 (1H; d; 15.9) | H_8_ | C_6_; C_9_ |
| 8 | 130.6/CH | 6.40 (1H; d; 15.9) | H_7_ |  |
| 9 | 197.5/C | - | - | _ |
| 10 | 28.6/CH_3_ | 2.24 (3H; s) | - |  |
| 11 | 23.2/CH_3_ | 1.04 (3H; s) | - | C_1_; C_2_; C_6_; C_12_ |
| 12 | 24.6/ CH_3_ | 0.96 (3H; s) | - | C_1_; C_2_; C6; C_11_ |
| 13 | 18.9/CH3 | 1.82 (3H; d; 1.2) | H_4_ | C_4_; C_5_; C_6_ |

* signal observed only in the spectrum gHMBC; NOESY: 6.77 (H_7_) × 2.44 (H_2a_) and 6.77 (H_7_) × 1.04 (H_11_).

**Figure 6S.** ^1^H-NMR spectrum of compound **6** (CDCl_3_/CD_3_OD, 300 MHz).

**
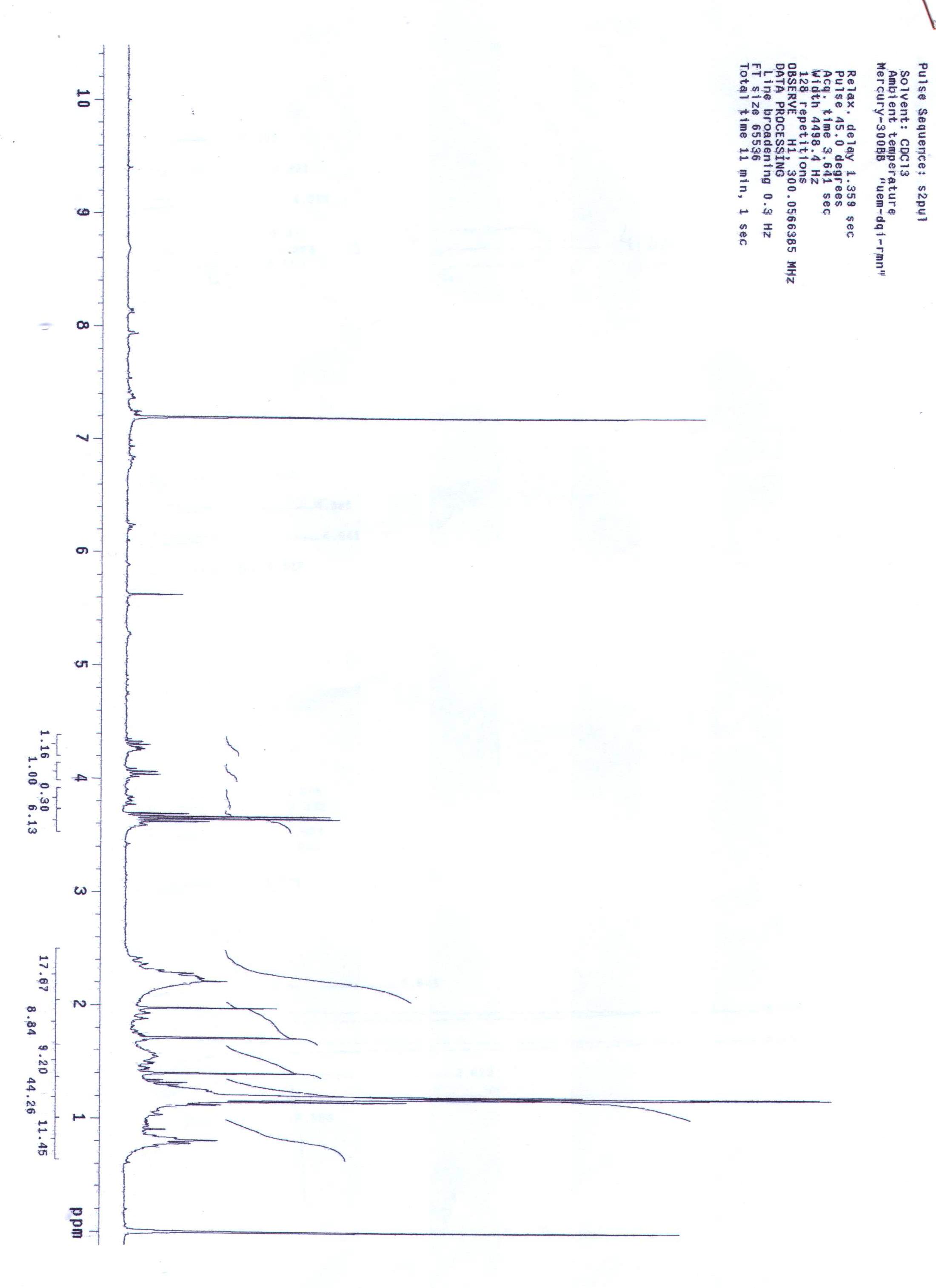
**

**Loliolide (6)**

**Table 6S.** Data of NMR (CDCl_3_/CD_3_OD, 300 MHz for ^1^H and 75 MHz for ^13^C) of loliolide (**6**) [15].

| **C** | **δ^13^C/DEPT** | **δ^1^H (nH; m, *J* = Hz)** | **gCOSY**  **^1^H×^1^H** | **gHMBC**  **^1^H→^13^C** |
| --- | --- | --- | --- | --- |
| 1 | *36.1/C | - | - | - |
| 2 | 47.6/CH_2_ | 1.47 (1H; dd; 14.4; 3.6)  1.91 (1H; dd; 14.4; 2.7) | H_2b_; H_3_  H_2a_ |  |
| 3 | 67.1/CH | 4.27 (1H; quint; 3.6) | H_2a_ | - |
| 4 | 45.9 /CH_2_ | 1.72 (1H; m)  2.39 (1H; dt; 14.1; 2.7) | H_4b_  H_4a_ |  |
| 5 | *87.0/C | - | - | - |
| 6 | *182.6/C | - | - | - |
| 7 | 113.2/CH | 5.63 (1H; s) | - | C_8_ |
| 8 | 174.1/C | - | - | - |
| 9 | 30.9/ CH_3_ | 1.21 (3H; s) | - | C_6_; C_10_; C_1_; C_2_ |
| 10 | 26.7/CH_3_ | 1.40 (3H; s) | - | C_2_; C_9_; C_1_; C_6_ |
| 11 | 27.2/CH_3_ | 1.71 (3H; s) | - | C_5_; C_4_; C_6_ |

* Signals observed only in the spectrum gHMBC
